# Improvement of Water-Cooling Performance for Combustion Chamber Through Optimization of Flow Channel Structure

**DOI:** 10.3390/ma19010087

**Published:** 2025-12-25

**Authors:** Daijian Wu, Guozheng Quan, Fanxin Meng, Si Li, Yanze Yu

**Affiliations:** 1 Chongqing Key Laboratory of Advanced Mold Intelligent Manufacturing, School of Material Science and Engineering, Chongqing University, Chongqing 400044, China; 2 Sichuan Provincial Engineering Research Center of Advanced Manufacturing Technology of Ramjet Engines, Sichuan Polytechnic University, Deyang 618000, China

**Keywords:** fluid characteristics, thermal–fluid–structure coupling, CFD calculations, equivalent stress

## Abstract

A complex operating environment poses significant challenges to the design of ramjet combustion chambers as high-enthalpy wind tunnels and their associated high-temperature, high-pressure combustion chambers continue to advance. This study developed a thermal–fluid–structure coupling finite element (FE) model based on the computational fluid dynamics (CFD) numerical simulation method to simulate the service conditions of combustion chambers under varying structures. Subsequently, FE simulation results were used to study the influences of combustion chamber structure on fluid flow characteristics, variation in cooling water pressure, temperature and stress of a combustion chamber wall. The results showed that after cooling water entered the chamber as a stable jet, it impacted the wall surface and formed a bidirectional vortex flow, which then entered the cooling water channels. Modifying the slope of a cooling water channel can effectively reduce pressure within the combustion chamber. It is noteworthy that the inlet equivalent stress of a combustion chamber decreases with an increasing slope, whereas outlet equivalent stress increases correspondingly. Finally, through comprehensive analysis, the optimal slope of a cooling water channel was determined to be 0.3°. This work provides essential theoretical insights for optimizing the design of combustion chambers.

## 1. Introduction

A high-enthalpy wind tunnel is a type of combustion heated wind tunnel primarily used as ground test equipment for hypersonic aircraft aerodynamic and propulsion research [1,2,3,4]. With the rapid advancement of aerospace technology, research and applications in the field of high-enthalpy wind tunnels and their associated high-temperature, high-pressure combustion chambers have continued to be carried out and investigated [5]. As a core thermal component of the high-enthalpy wind tunnel, the combustion chamber is subjected to intense convective and radiative heat fluxes generated by high-temperature, high-pressure airflow during operation, in addition to the coupled effects of internal heat conduction. To mitigate an excessive temperature rise, a coolant flow channel is incorporated into the chamber wall to enable effective water cooling via a circulating water system. However, limited by traditional manufacturing capabilities, the internal flow channels of combustion chambers are typically designed with simple, regular geometries. This configuration hinders the achievement of a uniform cooling performance distribution. During operation, it is susceptible to inducing localized overheating and thermal stress-induced cracking, ultimately leading to structural failure of the shell due to the loss of strength and subsequent ablation damage [6,7,8,9]. Therefore, it is of paramount importance for us to extend the service life of combustion chambers and ensure the safe operation of high-enthalpy wind tunnels to optimize the flow channel structure of combustion chambers, enhance cooling performance, and achieve reliable manufacturing.

When combustion chambers are serviced under stable conditions, high-temperature air undergoes consistent heat transfer with walls. Therefore, the optimization of the flow path structure plays a critical role in enhancing cooling performance [10,11]. The heat transfer process in combustion chambers involves convective heat transfer from high-temperature air to a wall, radiative heat transfer, and conductive heat transfer through a wall, followed by convective heat transfer from a wall to the cooling water. When water flows through a cooling water chamber, the flow near the wall surface undergoes flow separation and vortex formation [12]. This dynamic disturbance effectively reduces the thermal boundary layer thickness and enhances heat transfer between the mainstream region and near-wall region [13]. As the high-temperature and high-pressure air flows over the lower walls of the combustion chamber, a significant heat exchange occurs, resulting in a coupled thermal–fluid–structural interaction among the three components. However, traditional flow channel structure designs often focus on either thermal or structural behavior while neglecting the coupled effects that can lead to non-uniform temperature fields, localized stress concentrations, and premature material failure [14,15]. Caccavale P. et al. [16] demonstrated that thermal–fluid–structural coupling strongly influences wall heat transfer and stress evolution, particularly in regions with high thermal loads and complex flow patterns. Liu et al. [17] revealed that inlet geometry and pressure gradients can significantly modify flow uniformity and heat flux distribution, thereby altering wall temperature and stress field. Bryk M. et al. [18] performed a coupled thermal–fluid–structural analysis of the fuel tank’s internal geometry and demonstrated that moderate structural modifications can effectively reduce peak stress while maintaining cooling performance. Chen et al. [19] demonstrated, under actual operating conditions, that a fully coupled multi-physics simulation can effectively capture the dynamic interactions among coolant flow, temperature rise, and wall deformation. Furthermore, the high precision and capability of forming complex structures inherent in selective laser melting (SLM) technology enable its application in the integrated manufacturing of combustion chambers with complex flow channels, thereby opening new avenues for optimizing flow channel structure designs [20]. Therefore, it is essential to accurately simulate the thermal–fluid–structural coupling behavior in the combustion chamber to guide the optimization of the flow channel structure, thereby enhancing the cooling uniformity.

The dynamic characterization of the cooling water’s fluid properties and the operational behavior of combustion chamber components during service is critical for the design and optimization of structures. The three-dimensional numerical simulation method for fluid flow in complex cooling water channels, recognized as an efficient tool, is widely employed in the analysis of thermal–fluid–structure coupling [21,22]. Feppon F. et al. [23] addressed the strongly coupled thermos–fluid–structural problem associated with shape sensitivity by employing finite element (FE) analysis to achieve accurate interface resolution and reliable predictions of local thermodynamic responses. Liu et al. [24] obtained the temperature and pressure field distributions through fluid–thermal coupling simulations and subsequently incorporated these fields as thermal and mechanical loads into the structural FE model to analyze the stress–strain response and cumulative deformation behavior of the combustion chamber. These methods are employed to reveal the dynamic variations of various field quantities in thermal–fluid–structural coupling simulations. Therefore, establishing an FE model capable of accurately representing the service conditions of a combustion chamber is a critical prerequisite for reliably simulating the thermal and hydraulic state of cooling water.

**Figure 1:**
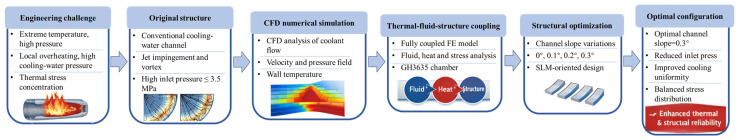
Schematic summary of the research process for combustion chambers.

This study systematically investigated the influence of various cooling water chamber geometries on the thermal–fluid–structure coupling behavior of a combustion chamber. Emphasis was placed on analyzing channel flow characteristics, pressure variations, and the effects of different chamber configurations on wall temperature distribution, internal pressure drop, and equivalent stress evolution within the combustion chamber. This study established a computational fluid dynamics (CFD) model to simulate heat transfer and fluid flow within a cooling water wall channel. The thermal–fluid–structure coupling process under varying combustion chamber structural parameters was analyzed, and the flow and heat transfer characteristics across different configurations were revealed. The summary of the process is presented in Figure 1. Firstly, a systematic study on the flow and heat transfer process of cooling water in a channel was conducted based on the CFD numerical simulation method. The vortex characteristics of cooling water within the combustion chamber cavity were successfully simulated, along with the temperature concentration zones on the original structural wall of workpiece. Subsequently, the pressure distribution of cooling water and stress distribution on the wall surfaces based on the optimized combustion chamber were studied. The results indicate that the cooling water pressure decreases with increasing slope of the cooling water channel, and the equivalent stress at the combustion chamber inlet decreases as the slope increases, while the equivalent stress at the outlet increases correspondingly. Finally, through comprehensive analysis, the optimal slope of the cooling water channel was determined to be 0.3°. This research can effectively reduce the water-cooling performance of combustion chamber flow channels. Valuable guidance is provided in these findings for the optimization and development of high-enthalpy wind tunnel combustion chambers.

## 2. Simulation and Experimental Methods

The service environment of the combustion chamber is characterized by a high level of complexity. This system involves heat transfer mechanisms comprising forced convection and radiation between high-temperature gas and a chamber wall, conductive heat transfer within the wall structure and forced convective exchange between the wall surface and the cooling water. This level of complexity is difficult to observe or measure directly through experimental methods. Currently, the numerical simulation approach of CFD has progressively become an essential tool in the study of flow and heat transfer phenomena [25]. In the process of developing a CFD model, the degree of integration between the FE method and the combustion chamber model, the selection of appropriate computational model, and the accuracy of the chosen model are all critically important factors.

### 2.1. Development of FE Model

**Figure 2:**
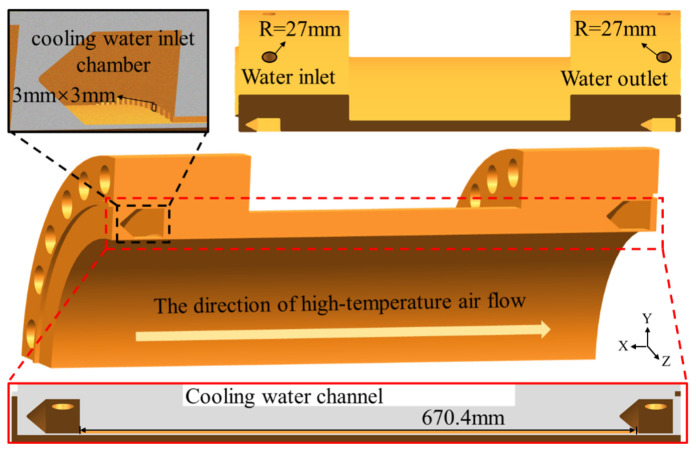
Physical model and dimensions of the quarter-combustion chamber.

This study focuses on a quarter combustion chamber as a research subject, with its physical model illustrated in Figure 2. The flow channel structure includes the water inlet, cooling water inlet chamber, cooling water channel, cooling water outlet chamber and water outlet. During the FE simulation process, the edge walls of the combustion chamber were designated as a periodic boundary condition. This not only substantially reduced the computational time but also maintained a high level of simulation accuracy. To ensure that the fluid reaches a fully developed state upon entering channel and to minimize the impact of outlet effect on calculation results, inlet and outlet sections were introduced at both ends of the high-temperature air and cooling water. Both the inlet and outlet sections feature smooth, insulated wall surfaces. This leads to both the inner walls of the high-temperature air and cooling water channels experiencing convective heat transfer with combustion chamber walls. However, a uniform heat flux density was imposed on all surfaces, excluding those in contact with the fluids. The size of the cooling water channel was 3 mm × 3 mm × 670.4 mm. The sizes of the inlet end of the high-temperature air channel and the cooling water channel were R = 420 mm and R = 27 mm, respectively. The size of the outlet end was identical to that of the inlet end. Based on the actual service conditions, the cooling water inlet was designated as a mass flow inlet. The quarter-combustor model was equipped with three cooling water inlets, each delivering a mass flow rate of 5 kg/s. The medium was liquid water at room temperature. The high-temperature air inlet was defined as a pressure inlet with a total pressure of 22 MPa. The medium was high-temperature air at 2400 K.

**Figure 3:**
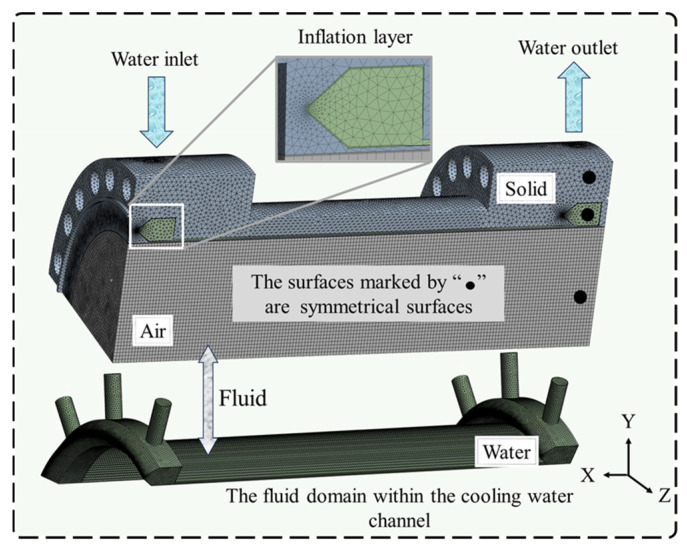
Schematic diagram illustrating the overall FE simulation model of the combustion chamber.

The material of the FE model for the combustion chamber is specified as a GH3625 [26] alloy, and its thermal physical property parameters are shown in Table 1. The overall FE simulation model of the combustion chamber is divided into three distinct sections: combustion chamber, high-temperature air, and cooling water. The model of the combustion chamber and meshing situation is shown in Figure 3. Separate meshing is carried out for different fluids and solids. At the fluid–solid interface, fine mesh refinement is applied on both sides to ensure that the coupling algorithm can accurately exchange thermal and mechanical loads. Generally, an increase in the number of meshes leads to higher accuracy in numerical simulation results. However, the issue comprising the computational workload also needs to be taken into account. Based on the verification of mesh independence with respect to the Nusselt number [27], a mesh configuration consisting of 2.8 million meshes was determined to serve as the reference mesh for study. In order to enhance the computational efficiency while ensuring calculation accuracy, hexahedral mapped mesh generation was utilized. Four layers consisting of an inflation layer were incorporated into the contact region between the fluid and solid to enhance the surface interaction. The present analysis was conducted under steady-state conditions, corresponding to the primary long-duration operating mode of the combustion chamber. In actual use, cooling water is supplied before high-temperature air, and the chamber rapidly enters a continuous steady regime. The high-flow water-cooling system effectively suppresses the temperature of wall fluctuations, thereby limiting the influence of transient thermal shocks during startup and shutdown.

In this study, a bidirectional coupling strategy was adopted, in which the fluid solver transfers pressure and convective heat-transfer loads to the solid domain, while the solid solver returns the updated wall temperature and thermally induced deformation to the fluid domain. This iterative exchange ensures convergence of the coupled fields and enables an accurate prediction of the interactions between coolant flow, heat transfer, and the structural response of the combustion chamber.

### 2.2. CFD Numerical Simulation Method

#### 2.2.1. Boundary Conditions and Governing Equations

Thermal–fluid–structure coupling is a complex process involving multi-field interactions. It is necessary to introduce certain assumptions into the model before conducting numerical simulations. This assumption facilitates the balancing of the calculation process and analysis of calculation results. The fluid is characterized as an incompressible ideal fluid, with its physical property parameters varying as a function of temperature. The effects of fluid volume forces were neglected in this analysis, such as centrifugal force and gravitational force. The flow and heat transfer characteristics within the channel are assumed to reach a steady state. The velocity slip and temperature slip on the wall surface are neglected in this analysis. The flow and heat transfer within the computational domain are considered to be continuous fields. Furthermore, the channel entrance serves as a mass flow rate inlet, and the fluid temperature at the entrance is 298 K. The corresponding expression is shown in Equation (1).

(1) $$x=0,u=w=0,m_{in}=5\;kg/s,T_{in}=298\;K$$

No-slip boundary conditions were applied to all wall surfaces. Moreover, the walls of both the inlet and outlet sections are defined as adiabatic, while a constant heat flux of *q* = 15,000 W/m^2^ is imposed on the outer wall of the test section.

The flow and heat transfer processes of fluids are governed by three fundamental principles: conservation of mass, conservation of momentum, and conservation of energy. By transforming these laws into their corresponding mathematical descriptions, the three fundamental control equations for the flow channel CFD calculation model can be derived [28]. The conservation of mass is shown via Equation (2).

(2) $$u\frac{\partial u}{\partial x}+v\frac{\partial u}{\partial y}+w\frac{\partial u}{\partial z}=0$$

The conservation of momentum is shown via Equations (3)–(5).

(3) $$u\frac{\partial u}{\partial x}+v\frac{\partial u}{\partial y}+w\frac{\partial u}{\partial z}=-\frac{1}{\rho }\frac{\partial p}{\partial x}+\frac{\mu }{\rho }\left(\frac{\partial^{2}u}{\partial x^{2}}+\frac{\partial^{2}u}{\partial y^{2}}+\frac{\partial^{2}u}{\partial z^{2}}\right)$$

(4) $$u\frac{\partial u}{\partial x}+v\frac{\partial u}{\partial y}+w\frac{\partial u}{\partial z}=-\frac{1}{\rho }\frac{\partial p}{\partial y}+\frac{\mu }{\rho }\left(\frac{\partial^{2}v}{\partial x^{2}}+\frac{\partial^{2}v}{\partial y^{2}}+\frac{\partial^{2}v}{\partial z^{2}}\right)$$

(5) $$u\frac{\partial u}{\partial x}+v\frac{\partial u}{\partial y}+w\frac{\partial u}{\partial z}=-\frac{1}{\rho }\frac{\partial p}{\partial z}+\frac{\mu }{\rho }\left(\frac{\partial^{2}w}{\partial x^{2}}+\frac{\partial^{2}w}{\partial y^{2}}+\frac{\partial^{2}w}{\partial z^{2}}\right)$$

The conservation of energy is shown via Equation (6).

(6) $$\rho c_{p}\left(u\frac{\partial T}{\partial x}+v\frac{\partial T}{\partial y}+w\frac{\partial T}{\partial z}\right)=\lambda \left(\frac{\partial^{2}T}{\partial x^{2}}+\frac{\partial^{2}T}{\partial y^{2}}+\frac{\partial^{2}T}{\partial z^{2}}\right)$$

where *u*, *v*, and *w* are the velocity components in the *x*, *y*, and *z* directions, respectively; *ρ* is the density of a fluid; *μ* is the dynamic viscosity of a fluid; *c*_p_ is the specific heat capacity of a fluid; *T* is the absolute temperature; *λ* is the thermal conductivity of a fluid.

#### 2.2.2. Selection of Turbulence Models

The choice of the turbulence model will have a direct impact on the accuracy of the simulation results. Four turbulence models were selected for the simulation of flow heat transfer in smooth wall channels, including the Standard k-ω model, SST k-ω model, RNG k-ε model, and Realizable k-ε model. The simulation results were compared with the calculated Nusselt number values and with an empirical friction-factor correlation in order to select the most appropriate turbulence model. The Reynolds number is a dimensionless criterion that characterizes the flow state of a fluid. The entrance Reynolds number [29] *Re* of a channel is defined via Equation (7).

(7) $$Re=\frac{\rho m_{in}D_{h}}{\mu }$$

where *ρ* is the density of fluid; *m_in_* is the inlet mass flow rate; *D_h_* is the hydraulic diameter of channel; and *μ* is the dynamic viscosity of fluid. The Gnielinski experimental correlation formula was utilized to determine the average Nusselt number for a smooth channel [30], as shown in Equation (8).

(8) $$Nu=\frac{\left(\xi /8\right)\times \left(Re-1000\right)\times Pr}{1+12.7\times \sqrt{\xi /8}\times \left(Pr^{2/3}-1\right)}$$

where *ξ* is the coefficient of resistance $\xi ={\left(1.82\mathrm{lg}Re-1.64\right)}^{-2}$; *Pr* is the Prandtl number of a fluid and $Pr=c_{p}\mu /\lambda$. The Petukhov experimental correlation formula can be utilized to determine the resistance factor for a smooth channel [31], as shown in Equation (9).

(9) $$f={\left(0.79\ln Re-1.64\right)}^{-2}$$

To ensure the accuracy of turbulence modeling for the cooling water channels, a turbulence model validation case was established prior to the full combustion chamber simulation. In this validation case, water is used as the working fluid, and the model represents a single cooling water passage. A mass flow inlet boundary condition is prescribed to ensure fully developed turbulent flow, with an inlet water temperature of 298 K. A constant wall heat flux of 15,000 W/m^2^ is applied to the heated section of the channel, while the inlet and outlet extension sections are treated as adiabatic to eliminate entrance and exit thermal disturbances. A pressure outlet boundary condition is imposed at the channel exit, and no-slip boundary conditions are applied to all solid walls. The flow is assumed to be steady-state, incompressible, and turbulent, with temperature-dependent fluid properties.

**Figure 4:**
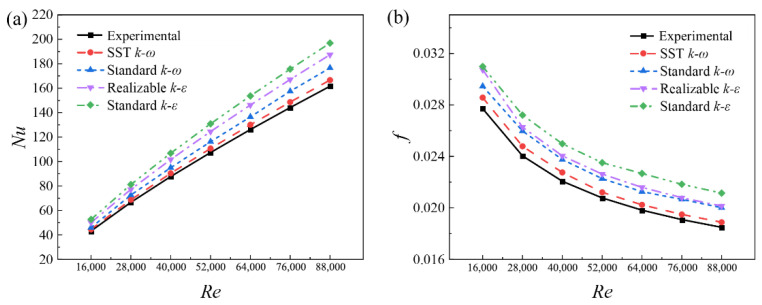
Comparison of simulated values with experimental correlations for different turbulence models: (**a**) Nusselt number. (**b**) Friction factor.

The Nusselt numbers and resistance factor obtained from various turbulence models were compared against the experimental correlation, as shown in Figure 4. The results indicate that the simulated values obtained from two k-ε models are substantially higher than calculated values using experimental correlation. The simulation results obtained using the SST k-ω model exhibit the closest agreement with experimental correlation. The maximum relative errors for Nusselt number and resistance factor are 3.10% and 4.48%, respectively. Therefore, the SST k-ω model was selected for the calculation and analysis of flow and heat transfer behaviors within the channel.

The SST k-ω model integrates the advantages of the k-ε and k-ω models. During the calculation process, the fluid domain is divided into a near-wall region and free-flow region. The k-ω model is utilized for computations in the near-wall region, whereas the k-ε model is utilized for calculations in the free-flow region. The transfer equation definition of SST k-ω [32] is shown in Equations (10) and (11).

(10) $$\frac{\partial }{\partial x_{i}}\left(\rho ku_{i}\right)=\tau_{ij}\frac{\partial u_{i}}{\partial x_{j}}-\beta^{*}\rho \omega k+\frac{\partial }{\partial x_{j}}\left(\left(\mu +\sigma_{k}\mu_{t}\right)\frac{\partial k}{\partial x_{j}}\right)$$

(11) $$\frac{\partial }{\partial x_{i}}\left(\rho \omega u_{i}\right)=\frac{\gamma }{v_{t}}\tau_{ij}\frac{\partial u_{i}}{\partial x_{j}}-\beta \rho \omega^{2}+\frac{\partial }{\partial x_{j}}\left(\left(\mu +\sigma_{\omega }\mu_{t}\right)\frac{\partial \omega }{\partial x_{j}}\right)+2\left(1-F_{1}\right)\rho \sigma_{\omega 2}\frac{1}{\omega }\frac{\partial k}{\partial x_{j}}\frac{\partial \omega }{\partial x_{j}}$$

where *μ_t_* is turbulent viscosity, and its calculation formula is shown in Equation (12).

(12) $$\mu_{t}=\frac{a_{1}\rho k}{\max\left(a_{1}\omega ,SF_{2}\right)}$$

The mixed function F_1_ is defined as Equation (13).

(13) $$F_{1}=\tanh\left\{{\left\{\min\left[\max\left(\frac{\sqrt{k}}{\beta^{*}\omega y},\frac{500v}{\omega y^{2}}\right),\frac{4\rho \sigma_{\omega 2}k}{CD_{k\omega }y^{2}}\right]\right\}}^{4}\right\}$$

The mixed function F_2_ is defined as Equation (14).

(14) $$F_{2}=\tanh\left\{{\left[\max\left(\frac{\sqrt{k}}{\beta^{*}\omega y},\frac{500v}{\omega y^{2}}\right)\right]}^{2}\right\}$$

The SST k-ω model transitions between the k-ε and k-ω models via the mixed function F_1_. In the near-wall region, F_1_ is equal to 1, while in the free-flow region, F_1_ is equal to 0. The parameter values in the SST k-ω model are derived from the k-ε and k-ω models. The two sets of parameters are integrated via mixed function F_1_, as shown in Equation (15).

(15) $$\varphi =\varphi_{1}F_{1}+\varphi_{2}\left(1-F_{1}\right)$$

where *φ* is a parameter value in the SST *k-ω* model; *φ*_1_ is a parameter value in the *k-ω* model; *φ*_2_ is a parameter value in the *k-ε* model. The specific values of each parameter in equations are shown in Table 2.

The numerical prediction results have _been_ compared with available combustion chamber test data, showing good agreement. Furthermore, the CFD results for a standard cooling channel closely follow the Dittus–Boelter correlation for the Nusselt number [33,34].

### 2.3. Coupling Mechanism of Thermal–Fluid–Structure Model

**Figure 5:**
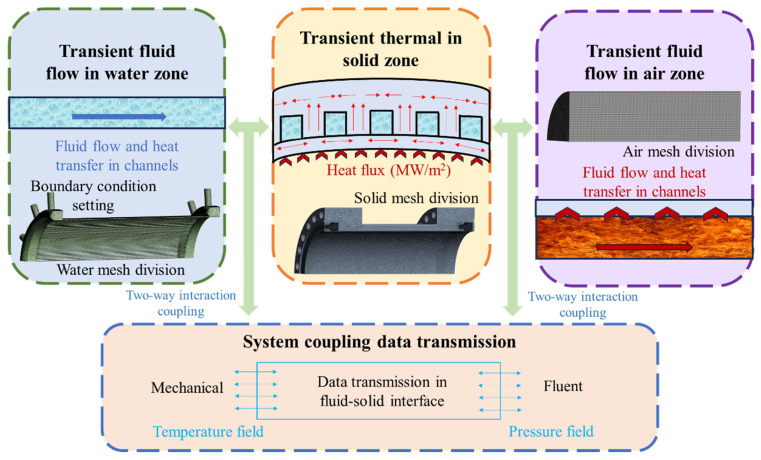
Coupling mechanism of thermal–fluid–structure model and data transmission.

The analysis of service conditions of the combustion chamber along with the computational workflow and data exchange within the thermal–fluid–structure coupling model are illustrated in Figure 5. The external wall surface adjacent to the combustion chamber is exposed to high-temperature air under elevated pressure, which acts as the primary heat source. Heat is first transferred from the hot gas to the solid wall and subsequently propagates through the solid structure by thermal conduction. On the opposite side, the inner wall surface of the cooling channel is in direct contact with water, forming the solid–fluid interface through which heat is removed by convective exchange. Furthermore, cooling water is introduced into the chamber prior to exposure to the high-temperature air, ensuring the simultaneous interaction of both fluids with the solid wall. The pressurized hot air and cooling water participates in thermal–fluid–structure coupling, and the distributions of thermal and flow fields are jointly determined by their interaction [35]. Based on the coupled fluid–solid interaction analysis, the results are utilized to determine the temperature and pressure field distributions. To accurately capture the dynamic changes occurring during the coupling process between surfaces, dynamic grid techniques were applied to the contact interface, thereby facilitating a precise evaluation of resulting responses.

## 3. Results and Discussion

### 3.1. Analysis of Combustion Chamber Service Conditions

The service environment of the combustion chamber involves forced convective and radiative heat transfer between the high-temperature gas and the chamber wall, conductive heat transfer within the wall structure, and forced convective heat transfer between the combustion chamber wall and the cooling water. The combustion chamber of the high-enthalpy wind tunnel experiences extreme thermal stress during operation: the gas-facing side is exposed to a maximum gas temperature of 2400 K and a pressure of up to 22 MPa, while the internal cooling channels must maintain effective water cooling. This study used a thermal–fluid–solid coupled FE analysis approach to examine the operational performance of the combustion chamber under conditions of high temperature, high pressure, and high heat flux density. The temperature, flow, and stress field distributions at critical locations were determined. Furthermore, the structural rationality and reliability were assessed, providing a design foundation for optimizing the flow channel configuration.

#### 3.1.1. Flow Characteristics of Water in the Cooling Flow Structure

Regular cross-sections, such as rectangles, trapezoids, or semi-circles, are commonly employed for cooling water channels in combustion chambers. Among these, rectangular channels are widely utilized due to their superior performance in both cooling efficiency and structural integrity. However, under the combined influence of high-pressure differentials and a sharp reduction in the inlet cross-sectional area, high-velocity jets tend to form at the inlet region, significantly altering the inherent flow structure within the channel.

**Figure 6:**
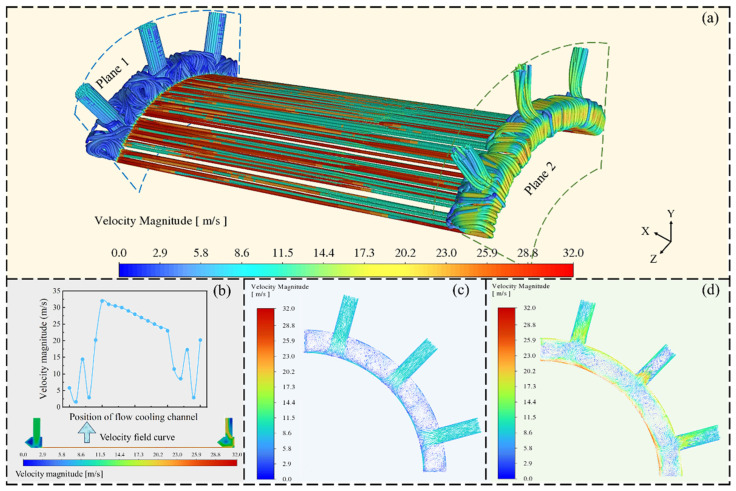
A schematic diagram of flow lines and velocity changes of the water in the cooling channel (**a**), velocity magnitude distribution along the flow channel (**b**), and fluid characteristic distribution of plane 1 (**c**) and plane 2 (**d**).

To systematically analyze the flow characteristics, this study conducted a comprehensive and systematic investigation of the flow field under the stable jet state, from the overall to the local level. Figure 6a illustrates the overall streamline distribution and velocity field within the cooling water passage. Figure 6b illustrates a quantitative analysis of the velocity amplitude variation along the passage. The results reveal extensive low-velocity regions in the inlet chamber and in the vicinity of the jet impingement zone. At the entrance of the rectangular channel, the fluid undergoes a sharp acceleration due to cross-sectional contraction and high-pressure driving, resulting in a localized jet core with a velocity reaching up to 32 m s^−1^. This spatially concentrated coexistence of extremely high and low velocities indicates the presence of intense shear flow and a jet-reflux structure within the water intake chamber.

To further elucidate the local flow structures arising from the high-speed flow field, Figure 6c illustrates a detailed depiction of the flow characteristics in a specific region. Upon entering the water inlet chamber, the cooling water impinges on the channel wall at a high speed, resulting in an abrupt change in flow direction from the initial radial orientation to lateral movement along the wall. The fluid flows between adjacent walls and undergoes multiple directional changes, ultimately leading to the formation of vortex structures on both sides of the impingement zone. This phenomenon is fundamentally attributed to the coupled effects of high-Reynolds-number flow, geometric discontinuity, and boundary layer development. The inherently high flow velocity and small hydraulic diameter of the system cause the inlet Reynolds number to exceed the critical threshold, leading to a significant reduction in flow stability under dominant inertial forces. Meanwhile, the abrupt contraction of the flow channel cross-section induces rapid fluid acceleration and flow separation, resulting in the formation of large-scale vortices in the near-wall region of the rectangular flow passage, particularly at the section immediately downstream of the inlet. Furthermore, the extremely thin and highly shear-dependent velocity boundary layer at the entrance exists in a non-steady state and naturally undergoes a transition as it propagates downstream. Subsequently, the flow field continues to develop along the channel, entering a relatively stable decay phase before exhibiting distinct multi-valley characteristics near the outlet chamber wall. These velocity fluctuations are closely associated with local flow separation, recirculation zone formation, and subsequent reattachment behavior induced by the channel’s internal geometric configuration. In the outlet region, due to the change in cross-sectional shape, the cooling fluid is discharged in a jet-like manner, generating additional vortices that cause a rapid increase in fluid velocity. The flow characteristics in this region resemble those observed at the inlet, as illustrated in Figure 6d.

The jets and vortices present in the combustion chamber cooling system can disrupt flow uniformity, preventing the cooling water from effectively covering the hot wall surface, thereby leading to localized overheating and potential material ablation. At the same time, these vortices significantly increase flow resistance and system energy consumption and may induce periodic pressure pulsations that contribute to surface fatigue cracking. This non-uniform velocity distribution highlights the strong coupling between the geometric configuration and fluid dynamic behavior. Therefore, optimizing the geometry of the water intake chamber and the rectangular flow channel is essential for achieving uniform velocity distribution, enhancing cooling performance, and ensuring structural integrity under operational conditions.

#### 3.1.2. Pressure and Temperature Characteristics of Flow Channel Structure

**Figure 7:**
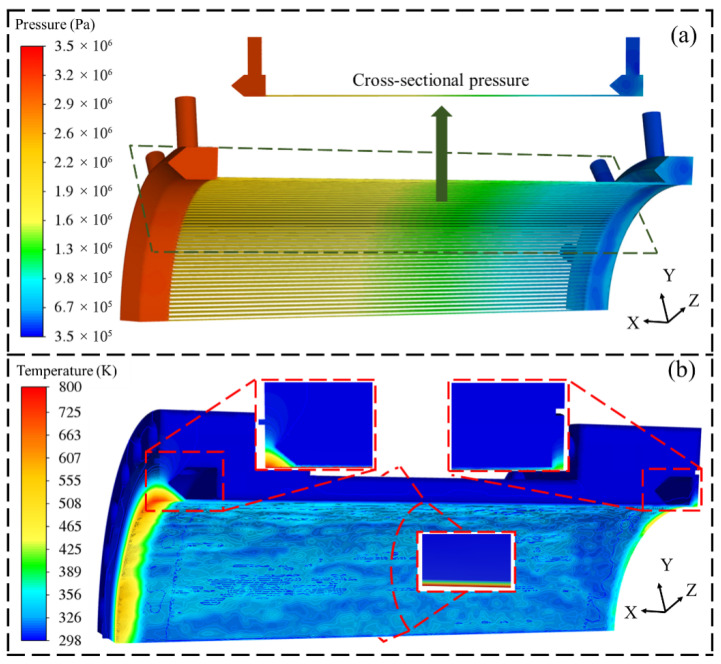
Cooling water pressure (**a**) and temperature distribution of combustion chamber (**b**).

Based on the numerical simulation results obtained from CFD, a comprehensive analysis was conducted to investigate the distribution characteristics of fluid pressure and combustion chamber temperature under a total cooling water flow rate of 60 kg/s. The system pressure decreases progressively from 3.4 × 10^6^ Pa at the inlet to 3.5 × 10^5^ Pa at the outlet, with the majority of the pressure drop occurring in the inlet chamber and the transitional section of the flow passage, as illustrated in Figure 7a. In the water inlet region, although the pressure does not exceed the upper limit of 3.5 × 10^6^ Pa specified in combustion chamber operating regulations, it approaches this threshold, indicating a limited safety margin in the current structural design. This high-pressure condition is primarily attributed to the combined effect of the pumping power and the stable vortex formed within the water inlet chamber. As the fluid transitions from the water inlet chamber into cooling water channels, the pressure remains between 2.2 × 10^6^ Pa and 2.9 × 10^6^ Pa along the mid-section of the channel. The pressure drop during this phase is predominantly governed by frictional losses along the flow channel. In the water outlet region, the pressure decreases to 3.5 × 10^5^ Pa, with static pressure further reduced due to increased flow velocity, reflecting the cumulative nature of total system pressure loss.

Figure 7b illustrates the temperature distribution characteristics within the combustion chamber, revealing three distinct regions of high-temperature concentration. This indicates systematic inadequacies in the current cooling structure when managing extreme thermal loads. First, in the triangular region between the high-temperature air inlet and inner wall of the cooling water channel, geometric constraints restrict coolant flow, resulting in sluggish fluid motion and low heat transfer efficiency. Consequently, the apex of this region becomes a persistent high-temperature zone. Second, in the 2 mm thick wall section adjacent to the cooling water channel, a continuous high-temperature band is observed on the wall surface despite uninterrupted cooling water flow, with local temperatures reaching up to 800 K. Furthermore, a pronounced high-temperature distribution is evident in the wall area corresponding to the water outlet chamber, indicating suboptimal cooling performance in this region. The presence of these three high-temperature zones highlights multiple limitations of the existing cooling design under localized thermal loading: specific geometrical configurations hinder uniform coolant coverage and convective heat transfer. Thin wall structures possess limited thermal capacity and struggle to maintain thermal equilibrium under sustained high-intensity heat flux, leading to a state of thermal saturation, and a non-uniform flow distribution across the system significantly reduces cooling effectiveness in critical areas. These thermal anomalies pose a direct threat to the thermal safety and long-term durability of the combustion chamber.

A comprehensive analysis reveals that the coexistence of an excessive overall pressure drop, insufficient pressure margin, and localized high-temperature regions on the wall poses significant risks to the safe operation of the combustion chamber. An excessive pressure drop not only diminishes the driving force of the cooling cycle but also compromises the ability of the system to thermally regulate high-temperature zones. Therefore, optimizing the geometric structures of the water inlet chamber and cooling water channels to reduce the inlet pressure, homogenize the pressure drop distribution, suppress local high temperatures, and improve the overall temperature uniformity is crucial for ensuring the efficient and reliable operation of the combustion chamber cooling system.

### 3.2. Design and Optimization of Combustion Chamber Structure Based on Service Conditions

The combustion chamber was manufactured using SLM technology. Optimizing the design of the cooling water inlet chamber and increasing the contact area between the cooling water and combustion chamber inner wall can significantly enhance the cooling efficiency. Furthermore, the maximum pressure is close to the critical threshold, placing the entire system in a state of operational instability during the combustion chamber service period. This is extremely detrimental to the service condition of a combustion chamber. When using SLM to fabricate such components, overhanging inner wall surfaces and complex structural features possibly lead to the formation of local overhangs, adversely affecting the dimensional accuracy of the final product. More importantly, the supports within the combustion chamber are difficult to remove and may cause structural damage during post-processing. Therefore, structural optimization requires design without requiring additional auxiliary supports during the shaping process.

The optimization performed in this study is a performance-driven geometric optimization of the cooling water channel configuration within the combustion chamber. A parametric comparative optimization strategy was adopted, which is suitable for complex thermal–fluid–structure coupling problems under realistic engineering constraints. The optimization objective is to enhance the service performance of the combustion chamber under normal operating conditions. The optimization constraints are defined by the actual service conditions and engineering limitations of the combustion chamber.

The optimization parameters focus on the geometric characteristics of the cooling water channel, which determines the inlet cross-sectional area and influences flow acceleration, pressure distribution, heat transfer, and structural loading. Four inlet slope configurations (0°, 0.1°, 0.2°, and 0.3°) were systematically evaluated. Their effects on coolant flow behavior, pressure levels, wall temperature distribution, and equivalent stress were compared to identify the optimal channel configuration.

#### 3.2.1. Design of Combustion Chamber Structure Based on Temperature Fields

**Figure 8:**
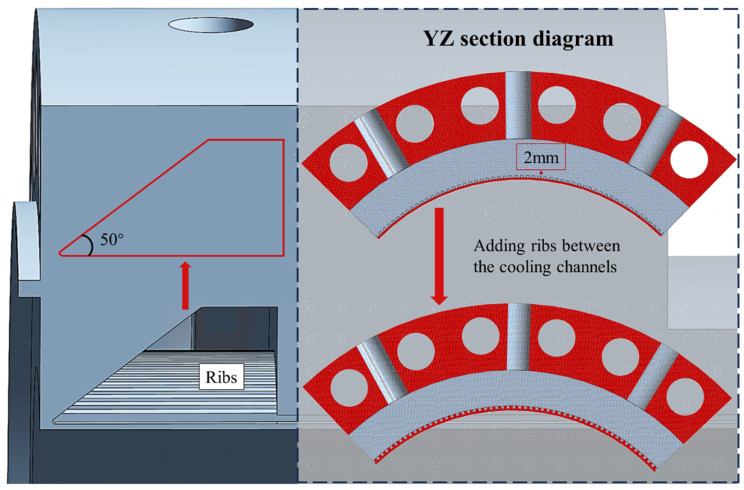
Schematic diagram of optimized cooling water inlet chamber structure.

The high-temperature concentrated zone at the air inlet of the combustion chamber renders it susceptible to oxidation and ablation during its service life. It is important to reduce the temperature concentration in this region. Based on the SLM molding process, the geometry of the water intake chamber was redesigned. The combustion chamber, following optimization of the cooling water inlet chamber structure, is shown in Figure 8. The triangular region of temperature concentration between the airflow and cooling water was minimized to the greatest extent. The inclination angle of the side wall of the water inlet chamber is 50°, which enables fabrication without the need for additional support structures. Furthermore, to mitigate the severe thermal load on the 2 mm thick wall directly exposed to the 2400 K hot gas, ribs were introduced between adjacent cooling channels. The addition of ribs increases the effective heat transfer area and induces secondary flow structures, such as flow separation and reattachment, thereby enhancing convective heat transfer on the coolant side. Meanwhile, the ribs act as conductive bridges that shorten the heat conduction path from the hot gas side to the coolant, which helps to reduce the wall temperature and homogenize its distribution. As a result, the temperature peak at the hot gas contact surface can be significantly suppressed, improving both the thermal safety margin and the service reliability of the combustion chamber.

**Figure 9:**
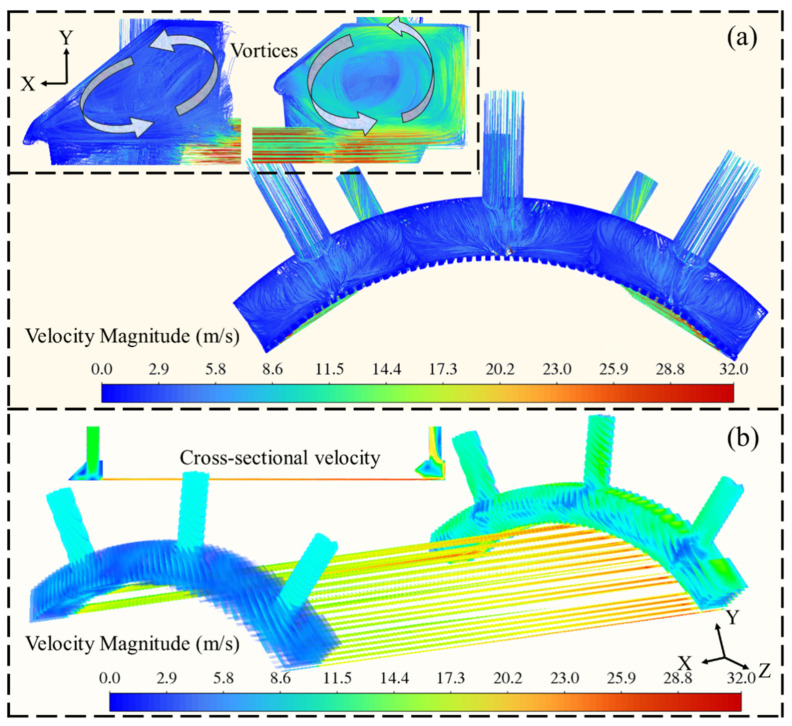
A schematic diagram of the optimized fluid characteristics (**a**) and velocity field distribution (**b**) in the combustion chamber.

Figure 9 illustrates the optimized flow characteristics of cooling water within the intake chamber and the corresponding velocity distribution in the combustion chamber. The flow characteristics of cooling water indicate that the structural modifications to side walls have a negligible effect on the generated vortices, as shown in Figure 9a. After modifying the inlet chamber geometry, the velocity distribution within the internal cooling channels remains nearly unchanged, as shown in Figure 9b. This is because the inlet mass flow rate and effective cross-sectional area are preserved, and the coolant is treated as an incompressible fluid. Consequently, the flow field automatically re-establishes a dynamic equilibrium between driving pressure and frictional resistance, leading to a consistent velocity pattern along the channel despite local geometric modifications.

**Figure 10:**
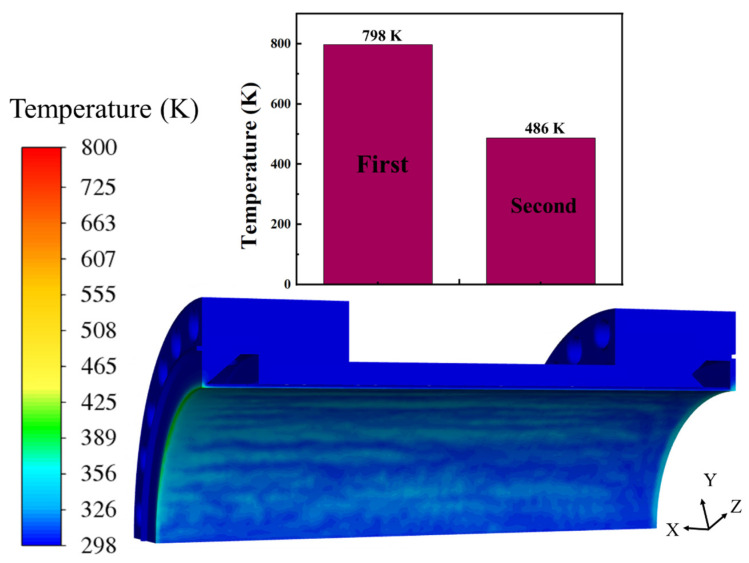
A schematic diagram of the temperature distribution in the combustion chamber after the water inlet chamber was optimized.

It should be noted that after the geometric modification of the inlet chamber, the temperature distribution within the combustion chamber exhibited a remarkable improvement, as shown in Figure 10. Compared with the initial configuration, the optimized structure significantly suppressed the high-temperature concentration near the lower wall adjacent to the hot gas region. The maximum wall temperature decreased by approximately 312 K, indicating a substantial enhancement in cooling efficiency. This improvement is primarily attributed to the redesigned flow channel geometry, which facilitates smoother coolant flow along the hot surface and strengthens the convective heat transfer near the inlet region where the heat flux is highest. Furthermore, the modified contour effectively promotes boundary layer disturbance and accelerates heat removal from the wall, resulting in a more uniform thermal field. Consequently, the previously localized hot spots were largely eliminated, and the overall wall temperature became more evenly distributed. These results confirm that the optimized chamber geometry achieves a better balance between thermal management and structural reliability under high heat flux conditions.

#### 3.2.2. Optimization and Analysis of the Internal Flow Channel Structure of Cooling Water

The FE simulation showed that the maximum pressure of the original structure was 3.5 × 10^6^ Pa. Although the design requirement specifies that the pressure in the cooling channels should remain below 3.5 × 10^6^ Pa, maintaining such a high level over an extended period can have adverse effects on both structural integrity and operational safety. Prolonged exposure to elevated internal pressure imposes continuous mechanical loading on the thin channel walls, which accelerates fatigue and creep damage, particularly in regions of geometric transitions. In addition, sustained high pressure reduces the safety margin against transient fluctuations caused by flow instabilities or thermal shocks, thereby increasing the likelihood of leakage or catastrophic failure. Maintaining a pressure at the cooling water outlet helps stabilize the flow field and ensures uninterrupted circulation within the cooling channel, thereby preventing flow disruptions. Furthermore, the steep geometry causes an uneven pressure gradient, leading to a non-uniform coolant residence time and reduced convective heat transfer in the downstream section. The intensified flow deflection imposes asymmetric loading on the thin channel wall, generating localized bending and thermal stresses that accelerate fatigue and creep under cyclic operation. From a hydraulic standpoint, an over-inclined channel also disrupts the pressure equilibrium between the inlet and outlet, increasing the risk of flow instability and compromising cooling consistency. From a system perspective, the outlet pressure also provides compatibility with the pump inlet conditions, reducing the risk of cavitation damage to the pump.

**Figure 11:**
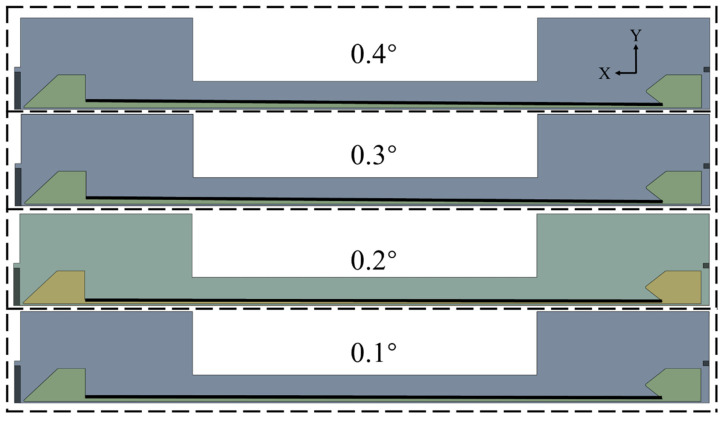
Optimization diagram of cooling water channel slope.

After entering the water inlet chamber, the cooling water flows in a swirling pattern into the cooling water channel within the chamber. Therefore, increasing the inlet slope and expanding the cross-sectional area of water inlet can effectively reduce the local flow velocity, weaken the inlet impact and flow separation, and minimize the vortices and secondary flows at the inlet. Figure 11 shows the optimized cross-section of cooling water channels on the XY plane of the combustion chamber and four different schemes for the inlet slopes of cooling channels. The cross-sectional areas of the inlets in these four schemes increase successively, whereas the cross-sectional areas of the outlets remain constant. Although the geometric change from 0° to 0.4° appears to be minor in terms of angle, it exerts a significant influence on the actual inlet cross-section due to the large overall size of the combustion chamber. For instance, the inlet height of the cooling water passage at 0.4° is approximately two to three times that at 0°. This design reduces local flow resistance and mitigates vortex formation at the inlet, thereby promoting a more uniform distribution of the coolant into the passage. At the same time, the constant outlet cross-section ensures that the overall mass flow rate and cooling capacity of the system remain unchanged.

**Figure 12:**
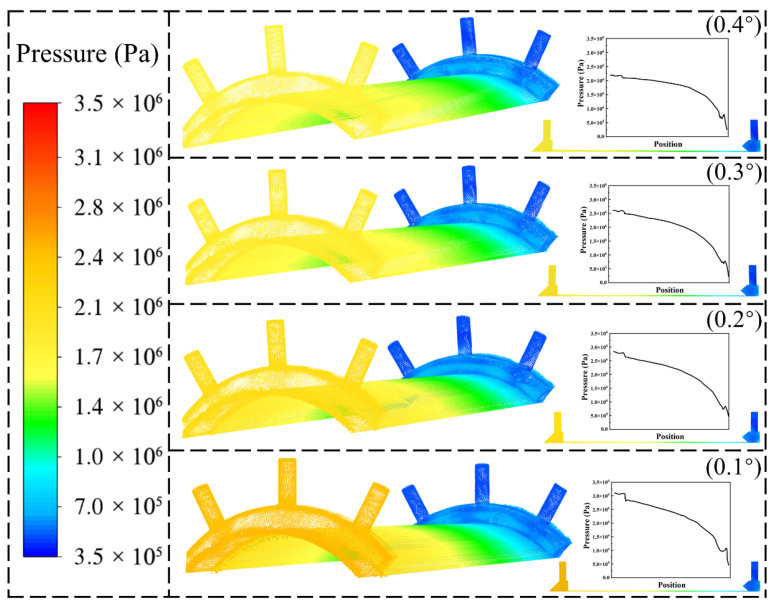
Pressure distribution diagram of the optimized strategy for different cooling water channels.

The pressure distribution within the cooling water channels after optimization is shown in Figure 12. In all cases, the pressure is highest at the inlet and gradually decreases along the flow direction, which is consistent with the expected behavior of incompressible coolant flow subject to frictional and local resistance losses. However, distinct differences are observed among the schemes. The total pressure level was the highest and reached a maximum of 3.2 × 10^6^ Pa when the cooling water channel structure had a slope of 0.1°, indicating a stronger driving force for cooling. As the structural slope increases to 0.2° and 0.3°, the pressure gradient distribution becomes more gradual, and the overall pressure level is moderately reduced. This suggests that, while maintaining adequate cooling capacity, flow uniformity is enhanced and hydraulic losses are minimized. In contrast, the 0.4° structure resulted in the lowest pressure of 2.4 × 10^6^ Pa among all the optimization schemes, alleviating mechanical stress. A small structural slope limits the inlet cross-section, which increases local resistance and accelerates the coolant at the entrance. This leads to a steeper pressure gradient and higher overall pressure levels, resulting in increased structural loading. Increasing the inlet area facilitates a smoother flow transition, suppresses flow separation and reduces local pressure losses in the vicinity of the inlet. Considering that the cooling water pressure inside the combustion chamber must remain below 3.5 MPa, an excessively small inlet slope is not favorable because it leads to a higher overall pressure level and increased structural loading. The appropriate structural slope can achieve a more favorable balance by ensuring a uniform flow distribution and adequate cooling performance while maintaining system pressure within allowable limits.

**Figure 13:**
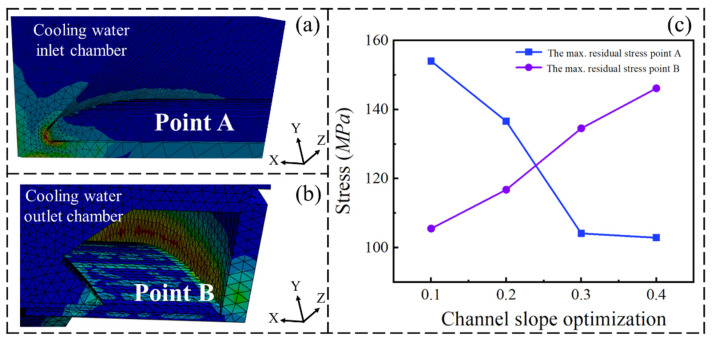
Equivalent stress distribution at the cooling water inlet (**a**), cooling water outlet (**b**) and equivalent stress variations under different cooling water channels’ optimization strategies (**c**).

The steady-state thermal–flow–structure interaction method is widely adopted. This method sequentially transfers the temperature field and pressure field to linear elastic structural analysis, thereby obtaining an accurate stress field [18]. The subsequent structural analysis was carried out using a linear elastic model, as the maximum operational stresses remain well below the yield strength of GH3625 and no material plasticity or creep is expected under steady-state conditions. Figure 13 illustrates the equivalent stress distribution at various locations within the combustion chamber under steady-state conditions of the cooling system during operation. The equivalent stress concentration regions are primarily located at the corners of a cooling water inlet chamber and along the flat sections of a cooling water outlet chamber, such as at points A and B. Figure 13c illustrates the equivalent stress values at points A and B under various optimization strategies with respect to the cooling channel slope. The results indicate that the equivalent stress value at point A decreases with an increasing slope. This trend is mainly attributed to a reduction in local inlet pressure and mitigation of the flow-induced equivalent stress concentration. The increase in channel slope reduces the fluid velocity and pressure in the inlet area. This smoother flow transition effectively reduces the hydraulic impact on the chamber wall, thereby alleviating the thermal–mechanical coupling effects that contribute to equivalent stress accumulation. However, the equivalent stress value at point B increases with an increasing slope. This behavior is primarily caused by the redistribution of fluid pressure and flow momentum along the channel as the inlet cross-section becomes larger. A greater slope leads to a reduction in inlet pressure but concentrates the driving pressure and flow acceleration toward the outlet region. The high-velocity cooling water flow impinges on point B. As a result, higher local pressure and intensified fluid impact occur near point B, which amplifies the thermal–mechanical coupling effects and induces greater structural equivalent stress.

The pressure–structure coupling analysis shows that cooling water channel slope optimization governs both the hydraulic driving force and the equivalent stress state of the combustion chamber wall. The small slope yields the highest overall pressure, which increases structural loading and elevates the inlet side equivalent stress. An excessive slope excessively lowers the channel pressure, weakening the cooling driving force and shifting equivalent stress toward the outlet side. By contrast, 0.2° and 0.3° slopes produce a smoother pressure gradient with pressure levels being kept below 3.5 MPa, enhance cooling water distribution, suppress wall hot spots, and reduce the peak equivalent stress at point A without an unacceptable rise at point B. Furthermore, as the slope increases from 0.2° to 0.3°, the equivalent stress at point A decreases by up to 32.45 MPa. The degree of equivalent stress reduction during this stage is the most significant. Meanwhile, the equivalent stress at point B increases by 17.78 MPa. This magnitude of change is similar to that observed when the slope increases from 0.1° to 0.2°. The results indicate that the service quality of the combustion chamber reaches its optimal level when the slope of the cooling water channel is 0.3°.

Through the structural optimization of the flow channels, particularly by adjusting the water inlet slope and cross-sectional geometry, the cooling performance of the combustion chamber was significantly improved. The optimization effectively reduced the water inlet pressure peak, resulting in a more uniform pressure gradient along the flow direction and a reduction of hydraulic losses within the system. This improvement promoted more stable coolant flow, enhanced convective heat transfer on the inner wall, and mitigated the formation of hot spots on the chamber surface. Additionally, the redistribution of pressure and flow momentum decreased the peak equivalent stress at critical inlet locations, thereby improving the overall structural reliability of the combustion chamber during high-temperature and high-pressure service conditions.

## 4. Conclusions

Numerical simulations were conducted for the operating conditions of the combustion chamber and the condition of the cooling water channels, while taking into account the transient thermal–flow–structural coupling effects. A comprehensive analysis of the interaction between fluid flow and the solid structure was conducted by examining the fluid flow characteristics and the temperature distribution within the solid. The main findings of this study are summarized as follows:

(1) The operating conditions of a combustion chamber were investigated, and the influence of the original structure on service performance was systematically analyzed. The geometry of a cooling water inlet chamber was designed and optimized, reducing the temperature at the highest concentration point of the combustion chamber.

(2) The fluid characteristics and pressure conditions within the combustion chamber cooling water system were systematically investigated. The cooling water flowed in a swirling pattern after entering the chamber and then slowed down as it passed through the channel. The pressure gradually decreased from the inlet to the outlet.

(3) The slope of cooling water channels was designed and optimized. The pressure decreased as slope increased, while the equivalent stress increased as the slope increased in the cooling water system. A comprehensive analysis indicated that the service condition of the combustion chamber reached its optimal state when the slope was set to 0.3°.

## Figures and Tables

**Table 1:** Thermal and physical property parameters of the GH3625 alloy.

Temperature (K)	Specific Heat (J·kg^−1^·K^−1^)	Thermal Conductivity (W·m^−1^·K^−1^)	Thermal Expansion (10^−6^ K^−1^)	Poisson’s Ratio	Young’s Modulus (GPa)	Yield Strength (MPa)
293.15	409.76	9.78	12.6	0.278	207.55	496.00
373.15	428.83	10.91	12.82	0.28	203.71	461.79
473.15	454.95	12.44	13.09	0.286	198.12	430.26
573.15	477.43	13.87	13.27	0.289	192.59	412.71
673.15	503.7	15.31	13.6	0.294	187.01	408.49
773.15	527.44	16.88	13.9	0.302	180.96	406.03
873.15	552.2	18.34	14.45	0.314	173.96	398.30
973.15	576.49	19.83	15.03	0.313	165.84	387.49
1073.15	600.81	21.52	15.48	0.316	155.88	330.55

**Table 2:** Parameters in the SST k-ω model.

Parameter	β*	*γ*	*β*	*σ_k_*	*σ_ω_*	*a*_1_
*φ*_1_	0.09	0.556	0.075	0.85	0.05	0.31
*φ*_2_	0.09	0.440	0.083	1.00	0.86	0.31

## Data Availability

The original contributions presented in this study are included in the article. Further inquiries can be directed to the corresponding author.
